# Clinical relevance of nine transcriptional molecular markers for the diagnosis of head and neck squamous cell carcinoma in tissue and saliva rinse

**DOI:** 10.1186/1471-2407-9-370

**Published:** 2009-10-18

**Authors:** Benjamin Lallemant, Alexandre Evrard, Christophe Combescure, Heliette Chapuis, Guillaume Chambon, Caroline Raynal, Christophe Reynaud, Omar Sabra, Dominique Joubert, Frédéric Hollande, Jean-Gabriel Lallemant, Serge Lumbroso, Jean-Paul Brouillet

**Affiliations:** 1 Service d'ORL et Chirurgie Maxillo-faciale, Centre Hospitalier Universitaire de Nîmes, Place du Pr. Robert Debré, 30029 Nîmes Cedex 9, France; 2 Centre National de la Recherche Scientifique, Unité Mixte de Recherche 5203, Institut de Génomique Fonctionnelle, Montpellier F-34094, France; 3 Institut National de la Santé et de la Recherche Médicale, U661 Montpellier F-34094, France; 4 Université Montpellier 1, Montpellier F-34094, France; 5 Laboratoire de Biochimie, Centre Hospitalier Universitaire de Nîmes, Place du Pr. Robert Debré, 30029 Nîmes Cedex 9, France; 6 Service d'Information Médicale et de Biostatistique, Centre Hospitalier Universitaire de Nîmes, Place du Pr. Robert Debré, 30029 Nîmes Cedex 9, France; 7 CRC - Service d'Epidémiologie Clinique, Hôpitaux Universitaires de Genève, Rue Micheli-du-Crest 24, CH-1211 Genève 14, Suisse; 8 Service d'Anatomie et Cyto-pathologie, Centre Hospitalier Universitaire de Nîmes, Place du Pr. Robert Debré, 30029 Nîmes Cedex 9, France

## Abstract

**Background:**

Analysis of 23 published transcriptome studies allowed us to identify nine genes displaying frequent alterations in HNSCC (*FN1, MMP1, PLAU, SPARC*, *IL1RN, KRT4, KRT13, MAL*, and *TGM3*). We aimed to independently confirm these dysregulations and to identify potential relationships with clinical data for diagnostic, staging and prognostic purposes either at the tissue level or in saliva rinse.

**Methods:**

For a period of two years, we systematically collected tumor tissue, normal matched mucosa and saliva of patients diagnosed with primary untreated HNSCC. Expression levels of the nine genes of interest were measured by RT-qPCR in tumor and healthy matched mucosa from 46 patients. *MMP1 *expression level was measured by RT-qPCR in the salivary rinse of 51 HNSCC patients and 18 control cases.

**Results:**

Dysregulation of the nine genes was confirmed by the Wilcoxon test. *IL1RN, MAL *and *MMP1 *were the most efficient diagnostic markers of HNSCC, with ROC AUC > 0.95 and both sensitivity and specificity above 91%. No clinically relevant correlation was found between gene expression level in tumor and T stage, N stage, tumor grade, global survival or disease-free survival. Our preliminary results suggests that with 100% specificity, *MMP1 *detection in saliva rinse is potentially useful for non invasive diagnosis of HNSCC of the oral cavity or oropharynx, but technical improvement is needed since sensitivity was only 20%.

**Conclusion:**

*IL1RN, MAL *and *MMP1 *are prospective tumor diagnostic markers for HNSCC. *MMP1 *overexpression is the most promising marker, and its detection could help identify tumor cells in tissue or saliva.

## Background

Head and neck squamous cell carcinoma (HNSCC) is the sixth most common malignancy worldwide and every year an estimated 600,000 people are newly diagnosed. HNSCC accounts for about 10% of the total cancer burden in men [1]. It is often diagnosed at late stages in heavy alcohol and tobacco users and patients presenting advanced disease have a short overall survival with major post-therapeutic side effects. Despite ongoing efforts, no molecular markers have yet been validated as useful clinical tools for the early detection and management of this disease. Recently, the transcriptome of HNSCC was extensively probed by several groups using expression microarray technology. The goals were to identify the genes potentially involved in this pathology and to identify diagnostic and/or prognostic gene-expression signatures [2-27]. Although the heterogeneous designs of these studies (number of sample, tumor sites, tumor stages, microdissection of tissue samples, RNA extraction and RT protocols, microarray technologies or biostatistics analyses) probably explain the diversity of the results, there is nevertheless strong motivation to develop robust molecular diagnostic tests [28].

We reviewed 23 studies published between March 2000 and November 2007, in which expression profiles of HNSCC tumors versus their normal matched mucosa were compared. Despite the important methodological heterogeneity of gene expression studies 9 genes appeared to be repeatedly dysregulated, in at least nine of the 23 studies for each gene [2-27]. Four genes were up-regulated in HNSCC tissues (*FN1, MMP1, PLAU *and *SPARC*) and five were down-regulated (*IL1RN, KRT4, KRT13, MAL *and *TGM3*). Because these genes were recurrently found to be dysregulated whatever the tumor location and despite the important methodological heterogeneity of the gene expression studies, we hypothesized that these nine genes could be candidate as HNSCC-specific molecular markers. We therefore evaluated by RT-qPCR their expression level in 46 HNSCC samples and 46 healthy matched mucosa taken from an independent cohort of 74 patients consecutively diagnosed with primary untreated HNSCC in our institution. We assessed their tumor diagnostic values and looked at possible correlations with clinical data. The need for early detection in high-risk patients led several groups to develop techniques for identifying molecular markers in bodily fluid rather than in an invasive biopsy unsuitable for cancer screening. Saliva is an easily accessible diagnostic fluid for screening HNSCC [29]. In this study, we assessed the capacity of *MMP1*, one of the most relevant markers, to detect HNSCC tumor cells in saliva cells; this provided information on its utility as a non invasive diagnostic marker.

## Methods

### Patients and sample collection

All normal and tumor tissues, as well as saliva samples, were obtained from the Institutional Tumor Bank of the University Hospital of Nîmes, France. All samples were collected with the informed consent of the patients. This study was approved by the local ethics committee and the scientific board of the Tumor Bank.

Tumor tissue, normal matched mucosa and saliva were obtained from 74 patients consecutively diagnosed with primary untreated HNSCC between April 2005 and April 2007 in our institution. All HNSCC patients were Caucasian heavy smokers and drinkers. Additional saliva samples from 18 healthy controls were collected, between February and April 2008. This cohort of control cases was composed of two populations: 9 consecutive patients refereed to our institution by their physician for HNSCC screening, motivated by heavy drinking and smoking addictions; 9 consecutive patients without alcohol or tobacco addiction in their past history that consulted for otologic or rhinologic mechanical disorders. All patients underwent a head and neck examination to determine the absence or presence of HNSCC and to rule out any significant inflammatory lesion of the upper aero-digestive tract. Patients in the control group were statistically a little younger and with more women than in the HNSCC group (average age = 52.8 years for controls vs 58.8 years for HNSCC [p = 0.037, student test]; sex ratio = 0.38 for controls vs 0.11 for HNSCC [p = 0.029 2-sample test for equality of proportions]).

Tissue samples were collected by biopsy during diagnostic endoscopy and were immediately snap frozen and stored in liquid nitrogen. The matched non-malignant tissue was collected from the same anatomical site, as far as possible from the primary lesion for tumors crossing the midline or on the opposite side for well-lateralized tumors.

For saliva collection both HNSCC patients and control cases followed the same protocole. Subjects were asked to refrain from eating, drinking, smoking, or oral hygiene procedures for at least 1 hour before collection and carried out a 30-second oral rinse with 50 ml of NaCl 0.9% solution that was immediately centrifuged at 2600 rpm for 15 minutes at 4°C. The supernatant was discarded and the pellet was diluted into 1 ml of NaCl 0.9% and stored at -80°C before RNA extraction. The clinical and histopathological features of the populations are presented in Table 1.

**Table 1:** Clinical and histopathological features of the population: A) tissue sample population; B) saliva sample populations

***Table 1-A***				***Table 1-B***			
***Tissu samples (n = 46)***				***Saliva samples: Control population (n = 18)***			
	Median	Min	Max		Median	Min	Max
**Age**	56.7	41.2	77.7	**Age**	53	32	71
							
		**Qt**	**%**			**Qt**	**%**
**Sex**	*Woman*	4	8.7	**Sex**	*Woman*	6	33.3
	*Men*	42	91.3		*Men*	12	66.6
							
**Site**	*Larynx*	8	17.4				
	*Oral cavity*	8	17.4	***Saliva samples: HNSCC population (n = 51)***			
	*Hypopharynx*	7	15.2		**Median**	**Min**	**Max**
	*Oropharynx*	23	50	**Age**	59	41	83
							
**T Stage**	*1*	1	2.2			**Qt**	**%**
	*2*	11	23.9	**Sex**	*Woman*	6	11.8
	*3*	19	41.3		*Men*	45	88.2
	*4*	15	32,6				
							
**N Stage**	*0*	18	39.1	**Site**	*Larynx*	5	9.8
	*1*	4	8.7		*Oral cavity*	11	21.7
	*2*	23	50		*Hypopharynx*	8	15.7
	*3*	1	2.2		*Oropharynx*	27	52.8
							
**M Stage**	*0*	46	100	**T Stage**	*1*	6	11.8
	*1*	0	0		*2*	12	23.5
					*3*	14	27.4
**Grade**	*1*	13	28.3		*4*	19	37.3
	*2*	27	58.7				
	*3*	6	13	**N Stage**	*0*	17	33.2
					*+*	34	66.7
**Death**	*Yes*	21	45.7				
	*No*	25	54.3	**M Stage**	*0*	50	1.9
					*1*	1	98.1
**Follow-up**	*Controlled*	25	54.3				
	*Progression*	10	21.7				
	*Recurrence*	11	23.9				

Qt: quantity

%: percentage

### RNA isolation, quality control and cDNA synthesis

To obtain homogeneous and histologically well-characterized samples for RNA analyses, tissue samples were cut with a cryo-microtome into 50-200 slices of 9-μm thickness in RNase-free conditions. At least three frozen slices taken from the sample core were mounted on glass slides and briefly stained with eosin-hematoxylin for histopathological examination by an experienced pathologist (H.C.). Tumor tissue versus normal surrounding tissue percentage (T/N %) was determined for malignant samples. HNSCC samples with less than 30% tumor cells were excluded from the study. Tissue samples were not microdissected in order to include in the qPCR analysis not only the tumor cells, but also the surrounding stromal cells, which are known to have altered transcriptional activity during the carcinogenetic process [30]. Normal tissues had to be composed of both stroma and its surrounding normal epithelial layer, without any tumor cells, to be included in the study. Total RNA was extracted from the remaining tissue slices using the RNeasy Mini Kit (Qiagen, Courtaboeuf, France). Saliva rinse RNA extraction was carried out using the RNA Isolation Kit on a MagNa Pure Compact instrument (Roche, Meylan, France).

RNA quality control and quantification were carried out on an Agilent 2100 Bioanalyser using Total RNA Nano II Chips (Agilent Technologies, Massy, France). To limit the impact of possible RNA degradation on our results, we selected for further analysis, only tissue samples with total RNA concentrations >85 ng/μl and values of RNA integrity number (RIN) >6. and saliva samples with total RNA concentration >40 ng/μl and RIN value >4. These criteria were never responsible of sample removal as satisfying qualities could be obtained by a second or third extraction procedure when required.

After quality control of tissues and saliva of the initial cohort of 74 HNSCC patients, 46 matched pairs of tumor tissue and normal mucosa and 51 saliva samples were useful. For 23 patients we analyzed both tissues and saliva. For 23 patients we analysed tissues only and for 28 patients saliva only. From our initial cohort of 74 HNSCC patients, 46 matched pairs of tumor tissue and normal mucosa as well as 51 saliva samples were useful. The amount of tissue, either tumor or normal mucosa, was insufficient for 28 patients to carry out pathological analysis and mRNA extraction. Saliva collection missed for 18 patients and saliva collection procedures were inadequate for 5 other patients. Finally only 23 patients were analyzed both at the tissue and saliva level. No relation was found between sample removal and tumor stage.

After quality control, 1 μg of total RNA was reverse transcribed using M-MLV reverse transcriptase and oligo dT_14-16 _primers (Applied Biosystems, Courtaboeuf, France). Samples were incubated for 10 minutes at 65°C, cooled on ice for 5 minutes, and incubated with reverse transcriptase for 1 hour at 37°C. Reverse transcriptase was then inactivated by heating at 95°C for 5 minutes. The resulting cDNA were diluted 1:10 for tissue samples and 1:4 for saliva samples before being used as PCR template.

### Quantitative real-time PCR (qPCR

We quantified the mRNA expression of three housekeeping genes and the nine genes of interest by real-time RT-qPCR using the Light Cycler Fast DNA Master^plus ^SYBR green kit on LightCycler 480 (Roche, Meylan, France). Stringent primers sets were designed using Oligo 6 Software (MBI, Cascade, CO, USA). To avoid false detection of genomic DNA, although DNase was used for the extraction procedure, amplification was done on spliced regions of the genes. Gene references and primer characteristics are listed in Table 2. For each qPCR reaction we used 2 μl of the diluted cDNA, 1 μl of 10 μmol.l^-1 ^forward primer, 1 μl of 10 μmol.l^-1 ^reverse primer, 5 μl Light Cycler Fast DNA Master^plus ^SYBR green I and 11 μl PCR water, for a final volume of 20 μl. The PCR cycle conditions were set as follow: a preincubation step for 10 minutes at 95°C followed by 40 cycles for tissue cDNA and 53 cycles for saliva cell cDNA; each cycle included 15 seconds at 95°C, 15 seconds at 60°C, and 15 seconds at 72°C. The temperature transition rate was 20°C/second. A melting curve was generated by linear heating from 50°C to 95°C in 20 minutes with 10 fluorescence measures every 1°C. A negative control, with no template, and a positive inter-run control were included for each gene in each qPCR run. All measurements were performed in triplicate. Standard curve assays showed an efficient amplification >1.8 for all genes and the specificity was shown by a single peak at the expected temperature on melting curve analyses (Table 2). For each gene the inter-assay coefficient of variation of crossing point values was <10% (data not shown).

**Table 2:** Characteristics of the gene-specific qPCR assays

Gene name (synonym)	Access n° Gene ID	Gene location	Primer sequence 5'-3'	Amplicon size Melting T°c	PCR efficiency
**KRT4**	NM_002272	12q12-q13	f: tcaacaacaagtttgcctc	185	1.94
keratin 4	3851		r: gtcattgcccaaggtatcta	90	
**IL1RN**	NM_173841	2q14.2	f: cctgtcctgtgtcaagtctg	257	1.80
interleukin 1 receptor antagonist	3557		r: cgtcctcctggaagtagaat	90	
**KRT13**	NM_153490	17q12-q21.2	f: tctctgtcttgctggtctga	234	1.88
keratin 13	3860		r: atgaagaggagatgaaggaa	89	
**MMP1**	NM_002421	11q22.3	f: aaagacagattctacatgcg	237	1.92
matrix metallopeptidase 1	4312		r: tgcttcacagttctaggga	85	
**PLAU**	NM_002658	10q24	f: ggactacatcgtctacctgg	230	1.90
plasminogen activator, urokinase	5328		r: caaactggggatcgttatac	88	
**SPARC**	NM_003118	5q31.3-q32	f: ggtgactgaggtatctgtgg	245	1.85
secreted protein acidic cysteie-rich	6678		r: aggtcttgttgtcattgctg	90	
**TGM3**	NM_003245	20q11.2	f: cactctccaatggcagtagt	215	1.93
transglutaminase 3	7053		r: cataaagacgctatccacat	88	
**FN1**	NM_212482	2q34	f: tgacacttatgagcgtcct	234	1.81
fibronectin 1	2335		r: aaacacttctcagctatggg	86	
**MAL**	NM_002371	2cen-q13	f: ataaagccgcagtagaactt	181	1.95
mal, T-cell differentiation protein	4118		r: agagtaaacacagcacccac	84	
**ACTB**	NM_001101	7p15-p12	f: tggctggggtgttgaaggtct	238	1,89
actin, beta	60		r: agcacggcatcgtcaccaact	90	
**B2M**	NM_004048	15q21-q22.2	f: cagcgtactccaaagattca	240	1.99
beta-2-microglobulin	567		r: gaatgctccactttttcaat	90	
**RPS18**	NM_022551	6p21.3	f: agcttgttgtccagaccatt	187	1.84
ribosomal protein S18	6222		r: tgaggaaagcagacattgac	87	

T°c: Temperature

For the salivary assay, 50 cycles of the above-described qPCR procedure were carried out in order to detect very low concentrations of mRNA. As this number of cycle was extremely high in order to detect very low concentration of mRNA, the PCR specificity was controlled with care by melting curve analysis of the PCR products and by negative controls. Whatever, the highest Cp value observed was 44.2 cycles. The whole RT-qPCR procedure was carried out with and without RT enzyme, confirming the absence of contamination by genomic DNA during the automated saliva rinse RNA extraction process (data not shown).

### qPCR data analysis

values were automatically calculated by LightCycler 480^® ^Software using the second derivative method and were imported into qBase, version 1.3.5, a free software program for the management and automated analysis of qPCR data, for quantitative analyses [31]. Normal tissue cDNA of patient 5 was arbitrarily chosen as a calibrator for each gene, and for this sample the expression level was set at 1 for each gene. For each gene qPCR amplification efficiencies were calculated by qBase from standard curves and were applied in the quantification algorithm. Relative quantities were normalized by qBase to a geometric mean of the three housekeeping genes *ACT, B2M *and *RPS18*. Concerning the housekeeping gene set stability assessment, raw relative quantities were tested with geNorm software, version 3.4, for the combination of the three genes. geNorm algorithm calculates a gene expression stability measure M for a reference gene based on the average pairwise variation for that gene with all other tested reference genes [32].

### Statistical analysis

S-Plus 2000 software (TIBCO Software, Inc., Palo Alto, CA, USA) was used to perform the statistical analyses. The quantitative variables were described by median, minimum and maximum values and the qualitative variables were described by frequencies and percentages. Genetic markers were compared between HNSCC tissues and matched normal mucosa using a Wilcoxon test for paired data. Comparisons were considered significant when p < 0.05.

The ability of each gene to diagnose the tumor tissue (HNSCC diagnostic ability) was represented by a Receiver Operating Characteristic (ROC) curve and the corresponding Area Under the Curve (AUC). The ROC curves and AUC were assessed by a non-parametric method [33]. The optimal cut-off used to calculate both sensitivity and specificity, was defined as the cut-off minimizing the number of misclassified tissues.

The clinical relevance of HNSCC markers was analyzed by correlating the expression level and clinical-pathological parameters. For each gene, the expression level in HNSCC was compared according to stage T, stage N, histological grade and tumor tissue versus normal surrounding tissue percentage (T/N %) using a Kruskal-Wallis test. The Cox model was used to analyze global survival and disease-free survival. For survival tests, the median follow-up was 1 year and 3 months.

Since no data were available concerning the diagnosis values of our candidate markers, it was not possible to determine a statistically based number of sample. Then, we decided to include all patients with exploitable samples, available at our Institutional Tumor Bank at the time of analysis.

## Results

### Housekeeping gene stability

To date there is no published evidence to guide the selection of suitable housekeeping genes for the normalization of HNSCC RT-qPCR studies. Hence, we chose to normalize our qPCR relative ratio by the geometric mean of three commonly used housekeeping genes in cancer studies: *ACT, B2M *and *RPS18*. We validated this approach using geNorm software, version 3.4, which gave an expression stability measure based on the average pairwise variation of the three genes [32]. In our assay the stability value (M) of the association of *ACT*, *B2M *and *RPS18 *was M = 1.2. This M value, below the 1.5 arbitrary cut-off recommended by the software, means that our set of three housekeeping genes was appropriate for normalization.

### Differential gene expression between HNSCC and normal matched mucosa

**Figure 1:**
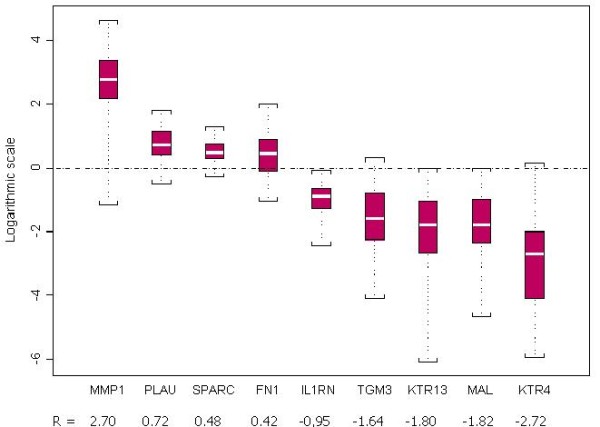
**Differential mRNA expression of the nine genes of interest in macroscopically healthy mucosa and HNSCC tissue**. For estimation of the individual expression of each gene, the expression ratios of paired tissue specimens were calculated as R = HNSCC/normal. The distribution of the log of these ratios is represented for each gene by a box-plot. The central box represents the interquartile interval, the white line inside the box is the median value, and the minimum and the maximum values are indicated with square brackets.

Normalized relative expression levels of the nine selected genes were calculated by qBase for each sample. These expression levels were compared between HNSCC and normal matched tissues for the 46 patients (92 samples). As presented in Table 3, relative mRNA expression levels were significantly higher in tumor than in healthy samples for *FN1, MMP1, PLAU *and *SPARC *(Wilcoxon test for paired data, p ≤ 0.002). These levels were lower in tumor than in normal samples for *KRT4, KRT13, IL1RN, MAL *and *TGM3 *(Wilcoxon test for paired data, p < 0.001). For each gene, the median expression level ratio between tumor tissues and their matched normal mucosa are presented in Figure 1. Among the overexpressed genes in tumor, *MMP1 *showed the highest order of magnitude (510-fold) and *FN1 *the lowest (2.6-fold). Among down-regulated genes in tumor, *KRT4 *showed the highest order of magnitude (530-fold) and *IL1RN *the lowest (9-fold).

**Table 3:** Expression levels of the 9 genes of interest in HNSCC and normal matched mucosa

Normal Tissue					***HNSCC Tissue***				
**Gene**	**median**	**St Dev**	**min**	**max**	***median***	***St Dev***	***min***	***max***	**p-values**

**FN1**	**0.076**	0.280	0.004	1.577	***0.268***	*0.471*	*0.007*	*2.185*	0.002
**IL1RN**	**1.801**	1.851	0.304	9.149	***0.204***	*0.304*	*0.010*	*1.675*	<0.001
**KTR13**	**9.110**	105.525	0.217	564.113	***0.156***	*5.193*	*<0.001*	*28.358*	<0.001
**KTR4**	**2.647**	75.657	0.080	479.146	***0.004***	*1.120*	*<0.001*	*6.003*	<0.001
**MAL**	**2.319**	3.378	0.262	19.968	***0.033***	*0.266*	*<0.001*	*1.182*	<0.001
**MMP1**	**0.755**	7.395	0.033	43.727	***319.480***	*1515.1*	*3.038*	*7510.565*	<0.001
**PLAU**	**0.995**	15.426	0.304	105.707	***6.831***	*18.192*	*0.274*	*109.053*	<0.001
**SPARC**	**0.302**	0.198	0.065	1.000	***0.985***	*0.996*	*0.140*	*4.791*	<0.001
**TGM3**	**1.399**	17.775	0.064	121.378	***0.035***	*13.594*	*0.001*	*92.326*	<0.001

The median, minimum and maximum values of relative normalized ratios for each gene of interest in normal tissue versus HNSCC tissue, with corresponding p-value (Wilcoxon test for paired data).

### HNSCC diagnostic ability of the nine genes

The ability of the nine selected genes to diagnose HNSCC was assessed by generating ROC curves. Corresponding AUC were calculated for each gene in order to find the best single marker to differentiate between normal mucosa and HNSCC tissue. Apart from *FN1*, which showed a poor AUC value (0.693), all genes demonstrated good diagnostic abilities with AUC > 0.86. Three genes were considered remarkable (*IL1RN, MAL *and *MMP1) *with AUC > 0.95. As presented in Table 4, when optimized cut-off values were selected, the single expression level of *IL1RN, MAL *or *MMP1 *correctly predicted the tumor or normal nature of tissue samples in, respectively, 87, 86 and 88 cases out of the 92 tested samples. Corresponding sensitivities ranged from 93.7 to 95.7% and specificities ranged from 91.3 to 97.8%. Interestingly, the association of these three genes in a multivariate model did not enhance diagnostic efficacy (data not shown). For each gene detailed statistics are listed in Table 4 (AUC of ROC curves, cut-off values and corresponding sensitivity, specificity, true positive value, false positive value, true negative value and false negative value).

**Table 4:** Diagnostic values of the 9 gene of interest

Gene	***AUC***	Cut-off value	False negative	False positive	True negative	True positive	***Sensitivity (%)***	***Specificity (%)***
**FN1**	*0,693*	0.20	19	11	35	27	*58.7*	*76.1*
**IL1NR**	*0,974*	0.51	3	2	44	43	*93.5*	*95.7*
**KTR13**	*0,921*	4.80	1	11	33	42	*75.0*	*95.5*
**KTR4**	*0,947*	0.23	5	4	42	41	*89.1*	*91.3*
**MAL**	*0,986*	0.72	2	4	42	44	*95.7*	*91.3*
**MMP1**	*0,989*	24.00	3	1	45	43	*93.5*	*97.8*
**PLAU**	*0,897*	2.30	9	5	41	37	*80.5*	*89.1*
**SPARC**	*0,864*	0.67	13	3	43	33	*71.7*	*93.5*
**TGM3**	*0,939*	0.31	7	4	42	39	*84.8*	*91.3*

Calculation of AUC values for the relative expression levels of the nine genes of interest by ROC analyses and the corresponding diagnostic values with optimum cut-off value.

### Clinical relevance of the nine genes

We investigated a possible association between the expression levels of the nine genes measured in tumor and T stage, N stage and histological grade. No statistical difference was found between expression levels in tumor of patients with stage N0 versus stage N+. The p-values ranged from 0.26 to 0.98 except for *KTR4 *(p = 0.08, Kruskal-Wallis test). Statistical differences were found between patients with histological grade 1 versus grade 2-3 for *IL1RN *(p = 0.01) and *SPARC *(p = 0.03). For the other genes, p-values ranged from 0.27 to 0.99. Statistical differences were found between patients with stage T2 versus stage T3-T4 for *SPARC *(p = 0.01). For the other genes, p-values ranged from 0.11 to 0.96.

A univariate survival analysis did not detect genes significantly correlated to global survival. For three genes, hazard ratios (HR) were low but with large confidence intervals: *TGM3 *(HR = 0.23 [0.02-2.94]), *MAL *(HR = 0.23 [0.02-2.24]) and *IL1RN *(HR = 0.38 [0.06-2.46]). Similar results were found when disease-free survival was analyzed.

### MMP1 mRNA detection in salivary rinse

Among the three best markers for HNSCC at the diagnostic level, *MMP1 *was the only one to be overexpressed in tumors. We hypothesized that its expression could be detected in the saliva rinses of patients with HNSCC, where tumor cells are known to desquamate. We carried out a preliminary assay to tested this gene in saliva but not the other 8 candidate genes. Among the 51 HNSCC patients, only 10 (20%) exhibited *MMP1 *mRNA at a measurable concentration in their saliva rinse. For these positive samples, *MMP1 *signal was detected at a mean Cp value of 36.4 cycles (31.7-44.2). As presented table 5, among the 10 HNSCC tumors detected by MMP1 salivary test, six aroused from the oropharynx, 3 from the oral cavity and 1 from the hypopharynx. MMP1 expression level in tissues was available for 3 out of the 10 patients with MMP1 positives salivary rinse. These three patients had 1000-fold overexpression of MMP1 in HNSCC comparing to normal matched mucosa. No *MMP1 *signal was detectable in the control group of 18 patients. *ACT, B2M *and *RPS18 *were used as positive internal control in this assay. Contrary to *MMP1*, their mRNA expression was detectable in all saliva samples at mean Cp values of 27.7, 28.8 and 31.7 cycles for *ACT*, *B2M *and *RPS18*, respectively.

**Table 5:** Characteristics of patients with positive MMP1 salivary test

Patient	Tumor site	T stage	N stage	M stage
*1*	*Oral cavity*	1	0	0
*2*	*Oral cavity*	4a	2b	0
*3*	*Oral cavity*	4a	2c	0
*4*	*Oropharynx*	2	0	0
*5*	*Oropharynx*	2	2b	0
*6*	*Oropharynx*	3	2b	0
*7*	*Oropharynx*	4a	0	0
*8*	*Oropharynx*	4a	2c	0
*9*	*Oropharynx*	4a	2c	1
*10*	*Hypopharynx*	3	2b	0

Tumor sites and TNM stages of the 10 patients with detectable levels of *MMP1 *in their saliva rinse.

## Discussion

Transcriptome profiling of tumor is a promising approach to identifying gene dysregulations potentially useful at the clinical level to detect or diagnose tumors and predict outcome, as well as to identify the gene pathways involved in carcinogenic processes. In the field of HNSCC, numerous studies have been performed but all have failed to find clinical applications, essentially because of the great diversity in their designs and the lack of a confirmatory step in an independent cohort of patients [2-27]. When we looked closely at the HNSCC transcriptome analyses some genes emerged as frequently dysregulated and therefore as specific candidates as HNSCC molecular markers.

This original study is the first to validate independently the gene dysregulation observed in various HNSCC transcriptome assays. Our study, based on RT-qPCR analysis, is not a definitive transcriptome validation assay, but it clearly proves that reliable biological information with potential clinical applications, can be obtained by pooling the results of several transcriptome data despite heterogeneous designs and small patient cohorts.

Using this gene selection and validation approach, we identified eight efficient transcriptional markers to predict the presence of HNSCC cells in tissue samples, with three remarkable markers: *IL1RN, MAL *and *MMP1*. When tested individually, these three markers presented specificity above 91% and sensitivity above 93% in a cohort of 46 patients with various stages, grades and sites of the disease. Surprisingly, there was no additional gain when these three top markers were evaluated together in a multivariate model rather than each separately. This finding implies that above 90% of both specificity and sensitivity little additional information can be expected by associating several transcriptional markers. It also indicates that the biological information associated with these three dysregulated genes should be somehow similar as far as diagnosis is concerned. It is worth mentioning that in clinical routine the use of only one well-characterized diagnostic marker has the advantage of being simple.

No clinically relevant correlations were identified between gene expression level measured in tumor and clinical or pathological parameters. This absence of correlation could signify that these dysregulations are common early events in HNSCC carcinogenesis, making these genes useful as diagnostic markers but useless as staging or prognostic markers. The proportion of early-stage tumor was low in our population, with 12 stage T2 tumors (26%); nonetheless, these nine dysregulations remained statistically significant in this subgroup of patient. This finding confirmed that these markers could be used for the detection of early-stage as well as later-stage tumors.

Among these three most remarkable dysregulated genes for HNSCC diagnosis, *MMP1 *was highly overexpressed. We thus hypothesized that it could be detected in the saliva rinses of patients with HNSCC for diagnosis or screening purposes. Indeed, several studies recently focused on the detection of such cells, mainly by studying DNA alterations (e.g., mutation, hypermethylation) but also by RNA detection [34]. Through a transcriptome salivary approach, Li et al. identified a set of seven overexpressed genes in saliva from patients presenting oral squamous cell carcinoma; their association in a multivariate model yielded a sensitivity of 91% and a specificity of 91% [35-40]. Li et al used the cell-free saliva for their transcriptome study. On the contrary, we choose to extract RNA from salivary floating-cells. In our opinion mRNA overexpression due to tumour alterations is more likely to be detected in the salivary floating-cells pellets than in the more RNA-diluted free cell saliva. Kim et al reported an elevation of *MMP1 *in saliva related to refractory periodontitis in microarray study of oral subepithelial connective tissues [41]. We do not think that periodontal health changes were responsible of *MMP1 *elevation in saliva of our patients since no clear differences of periodontal status were noticed between HNSCC patients and control cases or between *MMP1 *positive and *MMP1 *negative HNSCC patients.

As most of the patients likely to develop HNSCC are reluctant to consult physicians when the first symptoms occur, a noninvasive screening method based on the detection of tumor cells among epithelial oral cells could be of interest. In this preliminary salivary study, we confirmed that the expression of *MMP1 *was confined to HNSCC tumor cells as no expression was detected within the healthy population. Unfortunately, the sensitivity was low as only 20% of the patients presenting the disease were detected. Given that the salivary donors in our study presented advanced tumors, mostly symptomatic or easily detectable by standard clinical examination, this salivary approach could be considered as inefficient. On the other hand, the 100% specificity seems encouraging and HNSCC screening by a non-invasive salivary/oral screening test remains a promising field of research. In our study, the lack of sensitivity to detect salivary expression of *MMP1 *could have been due to suboptimal procedures for saliva collection, RNA extraction and retro-transcription. Sensitized procedures would increase the quantity of tumor cells and mRNA in samples and therefore improve the sensitivity of this technique [29].

The nine dysregulated genes have very different biological functions and some of them are already known to be implicated in cancer. From a clinical point of view, *IL1RN, MAL *and *TGM3 *were not clearly identified as potential HNSCC markers, while few data have been published concerning the implication of *FN1, KRT, MMP1, PLAU *and *SPARC *in this type of cancer. From a fundamental point of view, further studies are required to assess the functional mechanisms implicated in the dysregulations we observed.

## Conclusion

We confirmed in an independent study the dysregulation of *FN1*, *MMP1*, *PLAU SPARC*, *IL1RN*, *KRT4*, *KRT13*, *MAL *and *TGM3 *in HNSCC. Three of them, *MMP1*, *MAL *and *IL1RN*, were remarkable at identifying HNSCC in comparison with normal mucosa. They thus present an interesting potential as screening/diagnostic markers, still to be evaluated at the clinical level. *MMP1 *was the most promising gene of this study because it was overexpressed in tumor, highly dyregulated (500-fold) and tumor-specific. In addition to salivary detection, *MMP1 *could be tested at the RNA level for its ability to identify small tumor clusters in diagnostic biopsies, surgical margins or lymph nodes in the setting of primary tumors. This would be a valuable complement to histopathological analyses which efficacy for the diagnosis of microscopic tumors is low.

## Competing interests

The authors declare that they have no competing interests.

## Authors' contributions

BL was responsible for the conception, design and experimental work of the study and for drafting the manuscript. AE, CR, DJ, FH and SL made substantial contributions to the study conception and design and greatly enhanced the intellectual content of the manuscript. CC contributed substantially to the study design and carried out the statistical analysis. HC was responsible for pathological examination and quality control. GC, CR, OS and JGL carried out the tissue harvesting. JPB supervised the study and approved the final version of the manuscript. All authors read and approved the final manuscript.

## Pre-publication history

The pre-publication history for this paper can be accessed here:

http://www.biomedcentral.com/1471-2407/9/370/prepub
